# Effectiveness of Zinc Supplementation to Full Term Normal Infants: A Community Based Double Blind, Randomized, Controlled, Clinical Trial

**DOI:** 10.1371/journal.pone.0061486

**Published:** 2013-05-30

**Authors:** K. V. Radhakrishna, R. Hemalatha, J. J. Babu Geddam, P. Ajey Kumar, N. Balakrishna, Veena Shatrugna

**Affiliations:** National Institute of Nutrition (ICMR), Hyderabad, Andhra Pradesh, India; National Taiwan University Hospital, Taiwan

## Abstract

The study was aimed to test whether zinc supplementation, if initiated early, can prevent stunting and promote optimum body composition in full term infants. For this, full term pregnant women from low income urban community were enrolled and were followed-up for 24 months postpartum. Body mass index (BMI) was calculated from maternal weight and height that were collected one month after delivery. Infants' weight, and length, head, chest and mid upper arm circumferences and skin fold thicknesses at triceps, biceps and subscapular area were collected at baseline (before randomization) and once in three months up till 24 months. Three hundred and twenty four infants were randomized and allocated to zinc (163) or placebo (161) groups respectively. Supplementation of zinc was initiated from 4 months of age and continued till children attained 18 months. The control (placebo) group of children received riboflavin 0.5 mg/day, whereas the intervention (zinc) group received 5 mg zinc plus riboflavin 0.5 mg/day. When infants were 18 months old, dietary intakes (in 78 children) were calculated by 24 hour diet recall method and hemoglobin, zinc, copper and vitamin A were quantified in blood samples collected from 70 children. The results showed prevalence of undernutrition (body mass index <18.5) in 37% of the mothers. Mean±SD calorie consumption and zinc intakes from diets in infants were 590±282.8 Kcal/day and 0.97±0.608 mg/day respectively. Multiple linear regression models demonstrated maternal weight as a strong predictor of infants' weight and length at 18 months of age. As expected, diarrhea duration impacted infants' linear growth and weight gain adversely. Zinc supplementation for a mean period of 190 days, starting from 4 months up to 18 months of age, in full term normal infants, consuming an average energy of 590 Kcal/day, had significant effect on the skin fold thicknesses, but not on their linear growth.

**Trial Registration:**

Clinical Trail Registration India (CTRI) CTRI/2012/08/002884

## Introduction

It was shown earlier that zinc supplementation has significant effect on the linear growth of children, however, most of the studies were done in older children [1], [2]. The effect of zinc in younger infants is more important to document, since, linear growth restriction begins as early as 3–6 months of intrauterine life, continues through postnatal life and becomes irreversible by the age of 2 years [3], [4], [5]. Given that mild zinc deficiency is almost universal and considering the relatively higher requirement of zinc in early infancy and childhood, zinc should be considered as an important limiting factor to growth in children [6], [7], [8]. Complementary foods given to infants in India are predominantly cereal based and provide relatively inadequate amounts of zinc [6], [7], [8]. Higher physiological requirements of zinc for infants combined with low bio-availability from cereal based staple foods could be a reason for prevalence of mild to moderate zinc deficiency in developing countries like India [8], [9].

A meta-analysis of 33 prospective intervention trials showed significant effect of zinc supplementation on linear growth and body weight gain in children, whose mean age was 1.3 years [1]. Most of the studies were done in children after stunting became evident and the growth promoting response was found to be greater among stunted children compared to non-stunted children [1]. Moreover, a few studies that examined the effect of zinc, supplemented soon after birth were done among small for gestational age infants and the supplementation was given for less than six months [2], [10]. Since, stunting occurs even in full term infants, it would be interesting to study the effect of zinc on growth potential and body composition of full term infants.

Zinc is essential for lean body mass synthesis and its deficiency was shown to increase energy cost of tissue deposition [11]. Zinc deficiency was also shown to cause altered fatty acid metabolism leading to increase in fat mass, which eventually could lead to stunting associated obesity [11], [12].

The present randomized controlled clinical study aimed to investigate the effectiveness of zinc supplementation (one RDA) on growth and body composition of full term infants drawn from low income urban community.

## Methods

The protocol for this trial and supporting CONSORT checklist are available as supporting information; see Checklist S1 and Protocol S1.

### Ethics Statement

The study was approved by Scientific Advisory Committee (SAC) as well as the Institutional Review Board (IRB) of the National Institute of Nutrition (NIN). Written informed consent was obtained from the parents of all the participants. All clinical investigations were conducted according to the principles expressed in the Declaration of Helsinki.

### Study design and subjects

This study was a randomized, double blind, placebo controlled trial conducted in a low income urban community, which has a population of around 25,000 that is located in the Secunderabad city of the South India. Selection criteria included term healthy infants who would stay in the study area till the child attains 2 years of age. Preterm deliveries (gestational age <37 weeks), low birth weight (<2500 g) and infants with congenital abnormalities (neural tube defects, congenital heart disease, cleft palate and cleft lip) or birth asphyxia were excluded from the study. All the study children including other children from the community had access to a local medical center run by National Institute of Nutrition. However, conscious efforts were made not to interfere with the routine medical care provided either by the local health authorities through urban health centre or private medical practitioners. A clinic was conducted at the community center to assess study children and to treat any inter-current illnesses such as respiratory tract infections or dysentery; however, a few children who developed pneumonia were referred to tertiary care hospital.

### Sample size calculation

Assuming that the children in the experimental group would achieve better linear growth and that there would be ≥50% reduction in severe stunting compared to the placebo group, a sample size of 135 in each group was calculated taking the prevalence of severe stunting as 29.8 % in the age group of 1 to 2 years; with 80% power at 0.05 significance level with a corrected chi-squared test [13]. Expecting a 20% attrition, the total sample required was 160 per group. Power and Sample Size Calculation software, version 3.0.14 was used for sample size calculation.

### Subject recruitment

**Figure 1:**
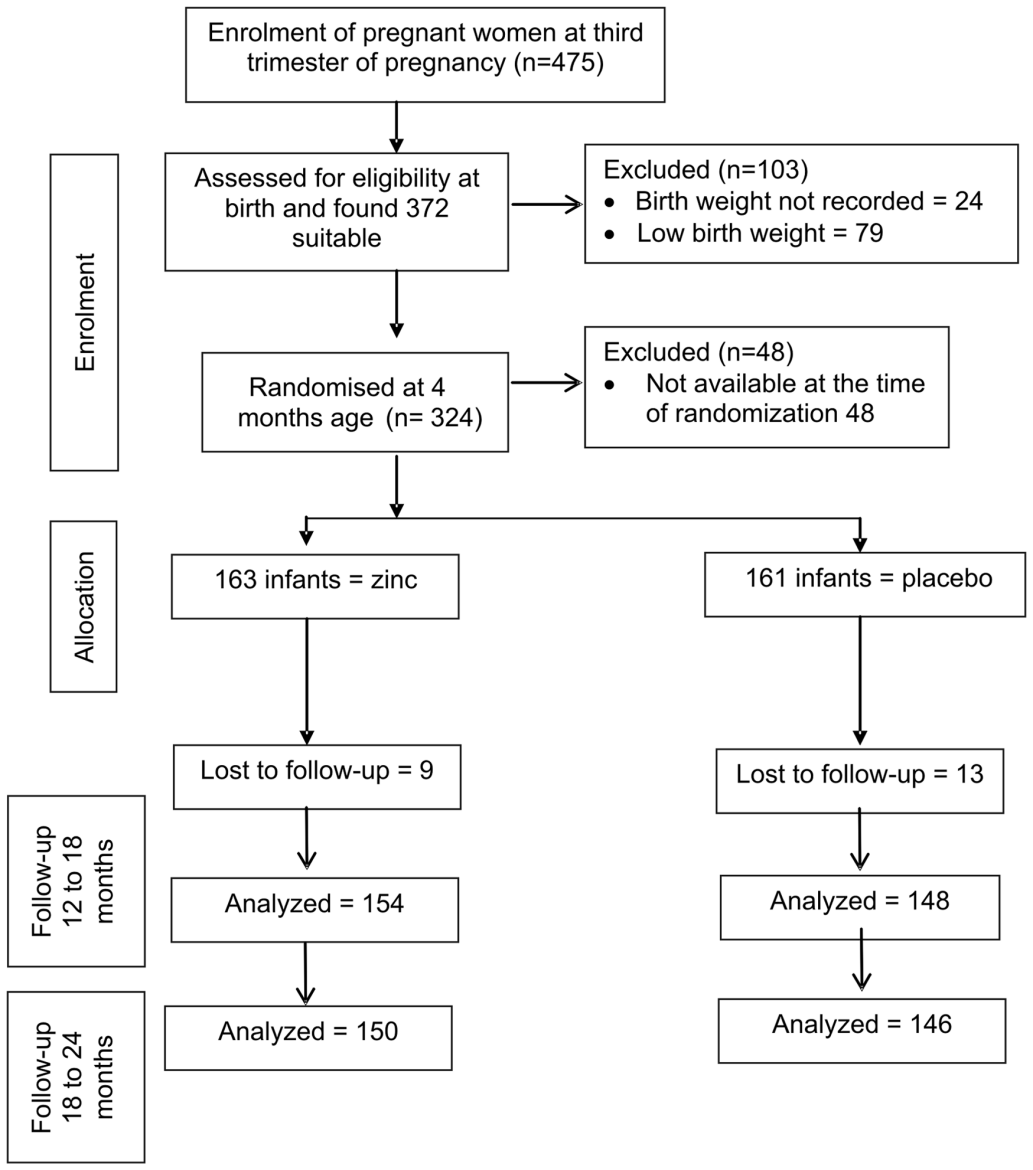
Flow diagram of the progress through the phases of the trial.

Four hundred and seventy five full term (gestational age ≥37 weeks) normal pregnant women were contacted and enrolled in the last month of pregnancy from a low income urban community. After delivery, new born babies were assessed for confirmation of eligibility and to identify potential noncompliant individuals who were not willing to participate. Of the 475 full term infants available, 24 did not have proper birth weight records and 79 were low birth weight babies and hence were excluded from the study. 48 infants were not available at the time of randomization as they moved out of the study area or refused to participate. Thus a total of 324 infants (163 and 161 for zinc and placebo groups respectively) were randomized (Figure 1). Supplementation was given after randomization to all children from four months of age till completion of 18 months. Mothers were counseled on strategies to overcome problems with supplement adherence. Eight and 14 children in zinc and placebo respectively, discontinued by 18 months, therefore in the final analysis only 302 were included (Figure 1).

### Randomization

The study was a double blind, randomized, placebo controlled trial, for which, a computer generated simple randomization was used to allocate the study children to either control or intervention group. The zinc and placebo were prepared and supplied by Biological Evans limited, in a syrup base, which were of similar colour, consistency and flavor; in two sets of identical looking bottles, labeled 1 and 2. Riboflavin (1 mg/ml) was common in both sets of bottles, but one set contained zinc as zinc sulfate (10 mg of elemental zinc per ml), in addition to riboflavin. The randomization list and the supplements were given to a senior scientist at the institute who had no knowledge of the codes. After recruitment, the study children were given an identification number and were assigned treatment code by the senior scientist supervising the randomization. All the investigators, including the medical doctor collecting clinical data and those collecting anthropometric measurement, as well as the statistician, were blind to the treatment. After completion of the study analysis, the groups were decoded. Thus, all the investigators involved in data collection, analysis and interpretation were blind to allocation. The parents were also blind to the treatment given to their child.

### Supplementation

Supplementation of zinc was initiated after the age of 4 months and was continued till the child attained 18 months age. The mothers were instructed to give 0.5 ml of the preparation to children every morning after the initial feed. The control groups of children received only riboflavin (0.5 mg/day), whereas the intervention group received 5 mg of zinc plus riboflavin (0.5 mg/day). Every month, the project staff, responsible for distributing the supplements in the field, collected empty bottles and provided fresh supplements as per subject identification number and codes.

The total duration of supplementation was for 14 months, however, supplements were not available for 2 months in between and none of the children received supplements during this period. Therefore the effective supplementation period was for 12 months (360 days) for each child.

### Data Collection

Data on nutritional status and morbidity of the children were collected by trained field investigators. Quality control testing for interrator reliability and reproducibility were done every three months. Data regarding feeding practices and any other supplementation were recorded for all the children. Immunization status of all children was recorded at monthly visits. Diarrheal and respiratory morbidity was collected by 15 day recall. Diarrheal episodes separated by 3 symptom free days were considered as separate episodes. Diarrhea was defined as passing of more than 3 stools in 24 hours or stools with altered consistency, with or without mucus or blood or loose watery stools. Respiratory tract infections were defined as cough or cold with fever and recurrence after 3 symptom free days was considered as a separate episode. Lower respiratory tract infections were confirmed by clinical examination and radiology.

Maternal weight and height were recorded 1 month after delivery, as most of the pregnancy weight gain is lost by one month postpartum. Birth weights were recorded from the delivery records. Anthropometric data collection like weight, length, head circumference, chest circumference and mid upper arm circumference and skin fold thicknesses at triceps, biceps, subscapular area were collected once in three months. Mothers were instructed to bring their children once in three months to the community center, which served as a central facility for anthropometric data collection. Whenever the children were not brought to the center, home visits were made and data recordings were done. In case the house was locked at the time of the visit, additional visits were made to record anthropometry measurement within the following two weeks. If they were not traceable within 2 weeks that particular recording was not done. Weight was recorded using a portable Seca digital weighing machine with a sensitivity of 50 grams and length was recorded using infanto-meter to the nearest of 1 mm. Skin fold thicknesses at biceps, triceps and subscapular were measured using Harpenden calipers. The children were categorized into different nutritional grades based on the WHO growth standard, 2006. Study children were defined as stunted, wasted or underweight if his or her ‘z’-score of weight-for-age (WAZ), length-for-age (HAZ) and weight-for-length (WHZ) respectively were <–2 SD. Children with ‘z’-score <–3 SD WAZ, HAZ or WHZ were classified as severely underweight, stunted or wasted respectively.

After completion of supplementation, when children were 18 months old, blood samples (3 ml) were collected from cubital vein in a sub sample of 70 children for estimation of Hemoglobin, Zinc, Copper and Vitamin A. Dietary intakes were also calculated by 24 hours recall method for 78 children (18 months old).

### Statistical analysis

Statistical software SPSS 19.0 was used for analysis. ANOVA and Mann-Whitney tests were done to test the differences between the groups. Other measures employed were repeated measures (time series) and Chi square to determine distribution of prevalence of undernutrition or stunting among zinc supplemented and control children at 18, 21 and 24 months of age. Regression analysis was done for determinants of infants' weight and length at 18, 21 and 24 months of age. The variables tested in regression analysis were gestational age, maternal weight, height, education and occupation; father's education and occupation and number of family members, for zinc and placebo groups separately as well as combined.

## Results

Maternal age, gestational age, birth weight, and other demographic variables at baseline were similar in both the zinc and the placebo groups (Table 1). Mean body mass index (BMI) of mothers was 19.8±3.09, with thirty seven percent being undernourished (BMI <18.5; Chronically Energy deficient, CED). Mean birth weight (kgs) was 2.86±0.34 and male to female ratio was 1.1: 0.9 (51.5% male and 48.5% females). At 3 months of age, before intervention, the mean weight, length, head circumference and mid upper arm circumference (MUAC) and the mean age of infants were similar in both the groups (Table 2).

**Table 1:** Demographic characteristics of the mothers and birth weight.

	Zinc (n = 163)	Placebo (control) (n = 161)	Total (n = 324)
Maternal age (years)	21.8±3.03	21.8±3.09	21.8±3.06
Gestational age (weeks)	39.7±0.63	39.6±0.63	39.7±0.63
Number of Ante natal visits	9.2±2.40	8.8±2.56	9.0±2.48
Father's age (years)	27.0±4.06	26.7±3.71	26.8±3.90
Number of family members	6.1±3.31	5.8±2.62	5.9±2.99
Maternal weight (kg)	45.6±7.30	46.8±7.89	46.2±7.60
Maternal height (cm)	153.1±6.13	152.7±6.15	152.9±6.15
Maternal BMI (kg/m^2^)	19.5±2.89	20.1±3.26	19.8±3.09
Birth weight (kg)	2.8±0.36	2.8±0.33	2.8±0.34

Values are Mean ± SD.

n  =  Number of subjects.

There were no significant differences between the groups.

**Table 2:** Baseline Infant characteristics at 3 months of age.

	Zinc (n = 163)	Placebo(control) (n = 161)	Total (n = 324)
Weight (kg)	5.5±0.73	5.5±0.77	5.5±0.75
Length (cm)	59.0±2.42	58.9±2.79	58.9±2.60
Head circumference (cm)	38.8±1.46	38.8±1.39	38.8±1.42
Mid upper arm circumference (cm)	12.3±1.12	12.4±1.17	12.4±1.14

All values are mean ± SD.

n =  Number of subjects.

There were no significant differences between the groups.

### Feeding pattern

In the first 3 months of life, >85% of infants were predominantly breast fed (breast feeding plus water) that declined gradually to 31.6% by 6 months. Around 10% and 75% of infants were partially weaned by 4 and 7 months respectively. By 18 months of age 19% were completely weaned. The proportion of infants that was exclusively breast fed and who were weaned at different time points were similar in both zinc and placebo groups. Similar proportion (61.6%) of infants in both zinc and placebo received some vitamin supplementation prescribed by regular practitioners/family doctors, with or without zinc in the first month of life, which gradually declined to 10.8% by fifth month. The mean±SD energy (Cal), protein (g/day) and zinc (mg/day) intakes, when children were 18 months old, were 590±282.8, 15.3±8.31 and 0.97±0.608 respectively, and were similar in zinc supplemented and placebo groups.

### Zinc supplementation

Though the effective supplementation period of zinc and placebo was 360 days for each child, the mean±SD duration of consumption of supplementation was 193.0±80.7 and 187.2±77.8 days for zinc supplemented and placebo groups respectively. Of the 163 and 161 infants randomized to zinc and placebo groups respectively, 9 in zinc and 13 in placebo were lost to followup by 18 months of age. Additionally, by 24 months of age, 4 and 2 infants were lost to followup in the zinc and placebo groups respectively. Thus, overall attrition was 8% and 9 % in zinc and placebo groups respectively (Figure 1).

### Overall nutritional status

**Figure 2:**
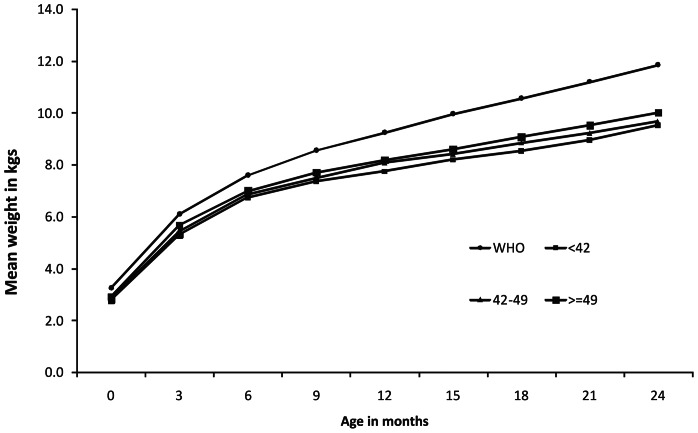
Mean weight of children from birth to 2 years by tertiles of maternal weight compared with WHO growth standards 2006.

**Figure 3:**
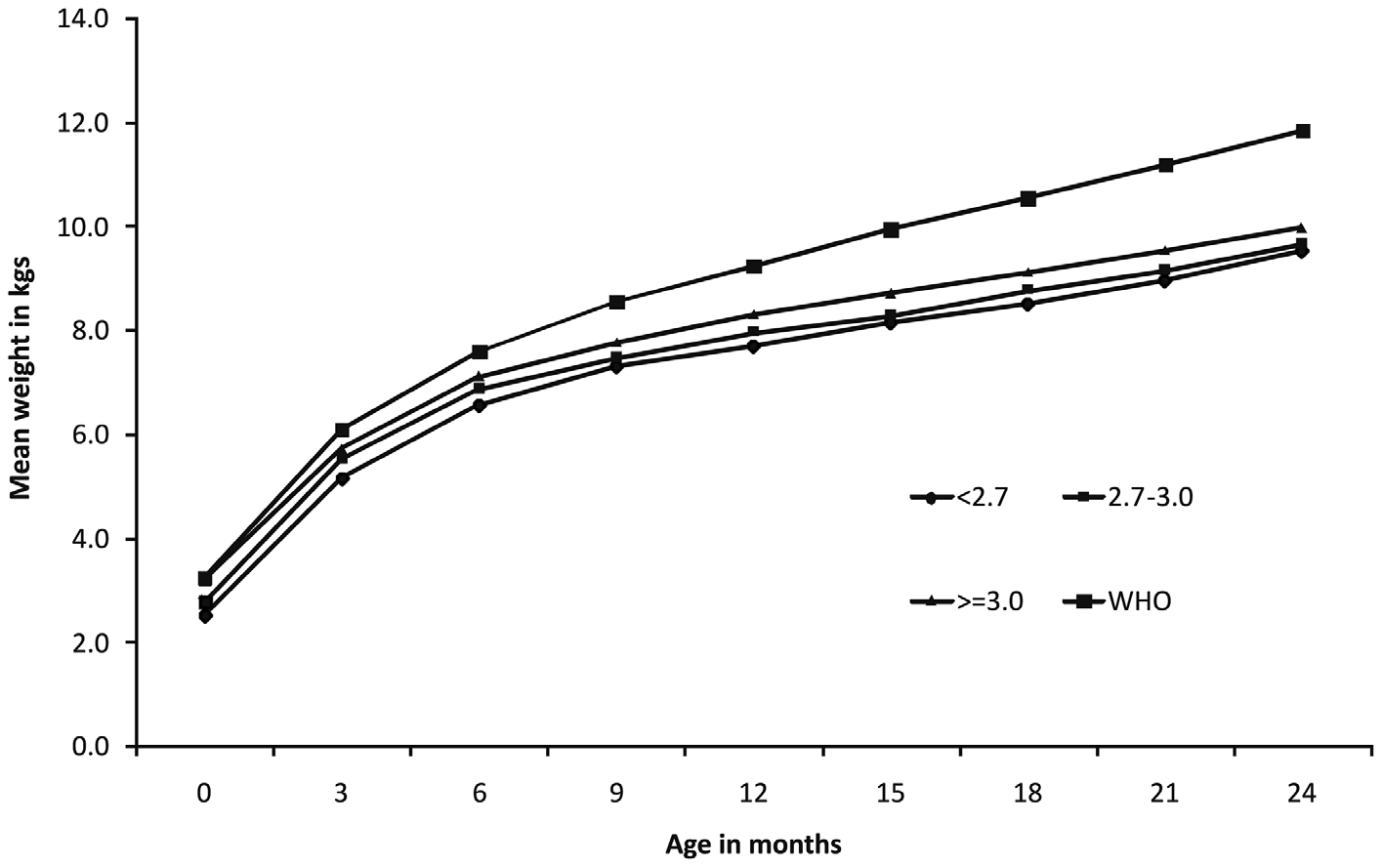
Mean weight of children from birth to 2 years by tertiles of birth weight compared with WHO growth standards 2006.

At 18, 21 and 24 months age, 38.4%, 37.8% and 35% infants were undernourished, 61.7%, 35.6% and 27.4% were stunted, 8.3%, 15.4% and 19.2% were wasted respectively (Table 3). When growth curves were plotted against the tertiles of maternal weight, irrespective of zinc supplementation, weight gain in children was directly related with postnatal maternal weight. Children born to mothers with highest tertiles were significantly heavier at all time points compared to those born to mothers in the lowest tertiles of weight or height or BMI (Figure 2 & Table 4). Similarly, cohort with the highest birth weight tertile showed better weight gain compared to the lowest birth weight tertile cohort (Figure 3).

**Table 3:** Prevalence (%) of underweight, stunting and wasting in study children.

Nutritional indicators	Age in months	Zinc	Placebo (control)	Total	P-value (by t-test)
Under weight (WAZ <–2SD)	18	38.4 (154)	38.4 (147)	38.4 (301)	1.00
	21	33.8 (152)	42 (147)	37.8 (299)	0.169
	24	33.1 (150)	37.0 (146)	35.0 (296)	0.504
Stunting (HAZ <–2SD)	18	61.6 (154)	61.9 (147)	61.7 (301)	0.962
	21	39.0 (152)	25.1 (147)	35.6 (299)	0.876
	24	29.5 (150)	25.2 (146)	27.4 (296)	0.432
Wasting (WHZ <–2SD)	18	6.5 (154)	10.1 (147)	8.3 (301)	0.268
	21	11.8 (152)	19.1 (147)	15.4 (299)	0.097
	24	14.3 (150)	24.6 (146)	19.2 (296)	0.033

Figures in parenthesis indicate number of subjects.

**Table 4:** Weights of study children by tertiles of maternal BMI.

BMI tertile	Less than 17.98 BMI (n = 107)	17.98 to 20.82 BMI (n = 109)	More than 20.82 BMI (n = 108)	P value by ANOVA
Birth weight (kg)	2.8±0.32	2.9±0.35	2.9±0.36	0.060
3 months	5.3±0.56	5.5±0.77	5.6±0.85	0.010
6 months	6.7±0.79	6.9±0.97	7.0±0.98	0.095
9 months	7.4±0.87	7.6±1.04	7.7±1.05	0.072
12 months	7.8±0.92	8.1±1.14	8.2±1.11	0.023
15 months	8.2±0.86	8.5±1.01	8.5±1.09	0.026
18 months	8.5±0.93	8.9±0.98	9.0±1.07	0.003
21 months	8.9±0.84	9.4±0.88	9.4±1.03	0.000
24 months	9.5±0.83	9.8±0.90	9.9±0.94	0.002

All values are Mean ± SD.

BMI: body mass index (kg/m^2^).

ANOVA: analysis of variance.

n  =  Number of subjects.

### Effect of zinc on growth indicators

**Figure 4:**
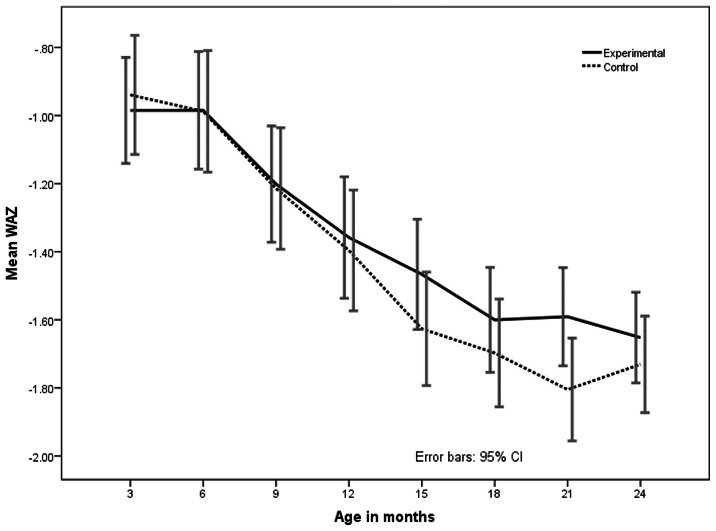
Mean Weight for Age z (WAZ) scores from 3–24 months in zinc supplemented (experimental) and control (placebo) children. The bars indicate 95% confidence interval.

**Figure 5:**
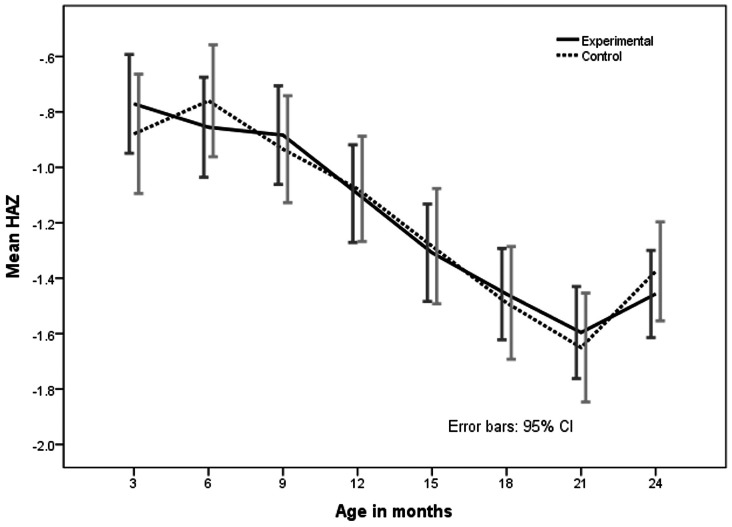
Mean Height for Age z (HAZ) scores from 3–24 months in zinc supplemented (experimental) and control (placebo) children. The bars indicate 95% confidence interval.

**Figure 6:**
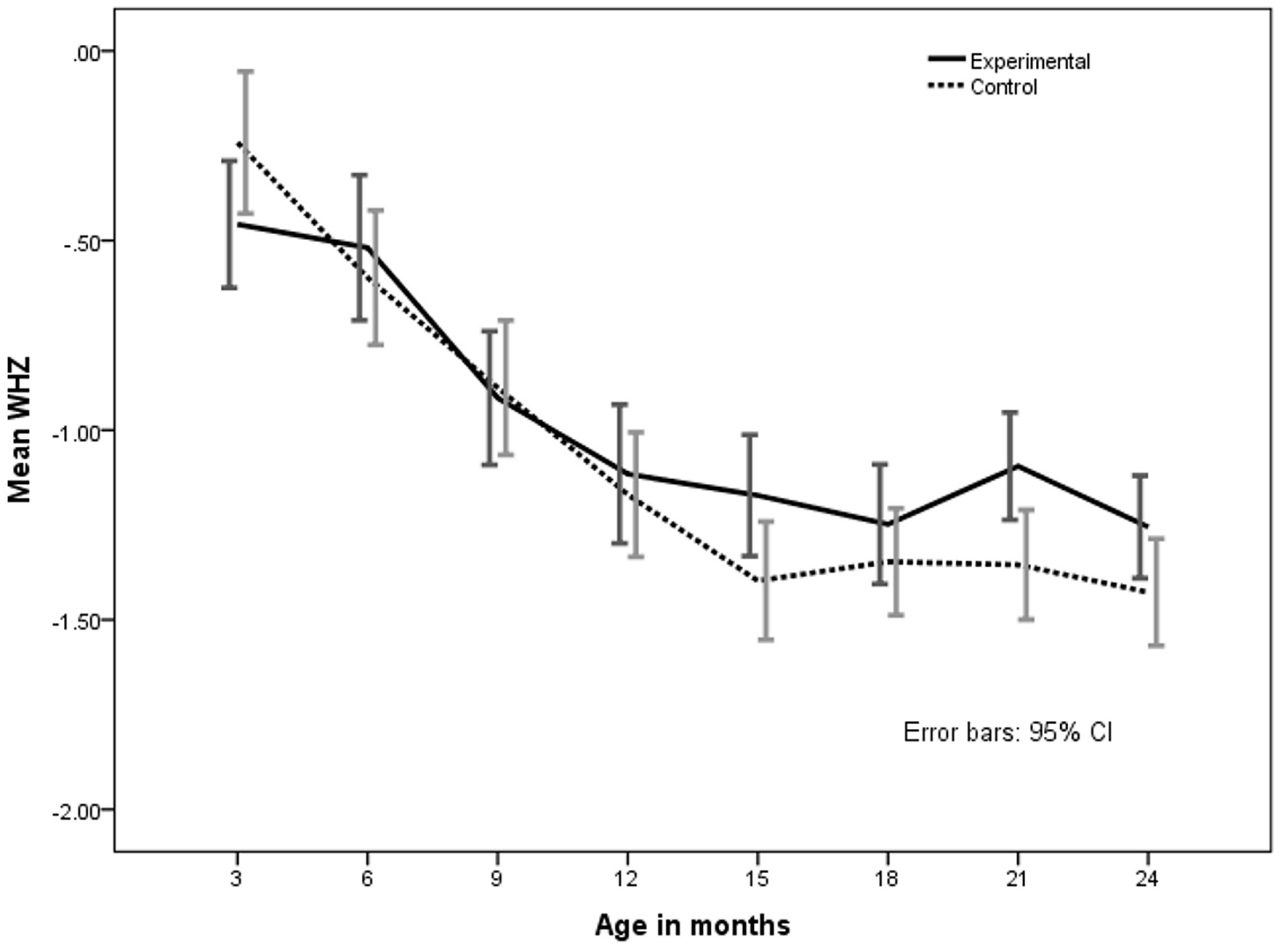
Mean Weight for Height z (WHZ) scores from 3–24 months in zinc supplemented (experimental) and control (placebo) children. The bars indicate 95% confidence interval.

On comparing placebo and intervention (zinc) groups, barring a notable decrease in the proportion of wasted infants in the zinc supplemented group at 24 months of age, there was no difference in the distribution of undernutrition, stunting and wasting between the zinc and placebo groups at 18, 21 and 24 months, (Table 3). Gain in length from 6 months to 18 months was 12.4 and 12.6 cm respectively, while weight gain was 1.92 kg and 1.98 kg respectively (Table 5). However, ‘z’ scores of weight for age (WAZ) and weight for height (WHZ) in the intervention group were better throughout the period from 6 months to 18 months, though there was no statistical significance (Figure 4 and 5). Height for age (HAZ) scores was not different at any given point of time between groups (Figure 6).

**Table 5:** Length and Weight of study children by age and groups.

Age in Months	Length (cm)		Weight(kg)	
	Zinc	Placebo (control)	Zinc	Placebo (control)
3	59.0±2.41	58.8±2.79	5.46±0.729	5.50±0.766
6	64.8±2.62	65.1±2.89	6.87±0.920	6.87±0.922
9	69.1±2.62	69.0±2.93	7.53±1.008	7.54±0.972
12	72.2±2.75	72.3±2.95	8.02±1.108	8.02±1.030
15	74.9±2.82	75.1±3.40	8.47±1.009	8.36±0.982
18	77.4±2.82	77.5±3.53	8.85±1.005	8.79±0.988
21	79.6±2.97	79.5±3.51	9.35±0.948	9.14±0.937
24	81.8±2.98	82.2±3.23	9.78±0.902	9.70±0.910

All values are mean ± SD.

There were no significant differences between the groups.

Predictably, skin fold thicknesses taken at three sites showed a significant increase in subscapular skin fold (SSF) and triceps skin fold (TSF) at 18, 21 and 24 months when compared to baseline. At 18 and 21 months age, the SSF was significantly (p = 0.022 and p = 0.033) higher in the zinc group compared to placebo group by mean of 0.331 (CI 0.049: 0.613) centimeter and 0.318 (CI 0.025: 0.611) centimeter respectively. Similarly, at 21 and 24 months the TSF was significantly (p = 0.011 and p = 0.024) higher in the zinc group compared to placebo group by mean of 0.425 (CI 0.095: 0.755) centimeter and 0.389 (CI 0.047: 0.731) centimeter respectively (Table 6).

**Table 6:** Skin fold thickness of the children during the study period from 3 to 24 months of age.

Variable	Number of subjects		Triceps skin fold thickness (mm)		Sub scapular skin fold thickness (mm)	
Age in months	Zinc	Placebo	Zinc (n = 163)	Placebo (control) (n = 161)	Zinc (n = 163)	Placebo (control) (n = 161)
3	n = 163	n = 161	8.2±2.06	8.2±2.13	7.1±1.53	6.9±1.40
6	n = 163	n = 161	8.2±2.26	8.1±2.25	6.9±1.77	6.6±1.56
9	n = 163	n = 161	8.2±1.84	8.0±1.77	6.8±1.54	6.5±1.34
12	n = 160	n = 158	8.1±1.70	7.8±1.55	6.5±1.33	6.4±1.19
15	n = 157	n = 154	7.7±1.43	7.7±1.36	6.5±1.29	6.3±1.16
18	n = 154	n = 147	7.8±1.37	7.6±1.30	6.4±1.36	6.1±1.07*
21	n = 152	n = 147	8.0±1.50*	7.6±1.19	6.5±1.35	6.2±1.05*
24	n = 150	n = 146	8.3±1.54*	7.9±1.26	6.6±1.27	6.4±1.10

All values are mean ± SD.

n  =  Number of subjects.

* P<0.05.

Stepwise Regression analysis was done for determinants of infant's weight and length at 18, 21 and 24 months of age. The variables tested were group, gestational age, maternal weight, height, education and occupation; father's education and occupation and number of family members. Maternal weight (β = 0.031; CI: 0.015, 0.047; p<0.001) and gestational age (β = 0.203; CI: 0.023, 0.383; p = 0.025) were contributing positively, while diarrheal duration (β = −0.027; CI: −0.046, −0.007; p = 0.006) was contributing negatively to infant's weight at 18 months of age. At 21 and 24 months of age, maternal weight (p<0.001) and gestational age (p = 0.005) emerged as strong predictors of infants' weight gain (Table 7). When regression analysis was applied at 18 months of age for determinants of length; maternal weight (β = 0.075; CI: 0.023, 0.126; p = 0.005) and father's education (β = 0.573; CI: 0.166, 0.979; p = 0.006) were contributing positively, while diarrheal duration was observed to influence negatively (β = −0.064; CI: −0.129, 0.001; p = 0.05). At 21 and 24 months of age, only maternal height (p<0.001) and fathers education (p = 0.01) emerged as strong predictors of infants' linear growth (Table 7). Even after adjusting the other variables zinc had no effect on linear growth, though, it contributed significantly for weight gain at 21 months of age (Table 7). Similarly, multivariate logistic regression model for stunting as dependent variable, at 18 months of age, revealed only a trend with maternal height (OR = 1.287; CI: 0.922, 7.532; p = 0.070), but at 21 months maternal weight was significantly associated with stunting (OR = 2.114; CI: 1.202, 3.718; p = 0.009). At 24 months of age, maternal height and illiteracy of father were significantly associated with stunting. ((OR = 2.895; CI: 1.139, 7.356; p = 0.025 and OR = 2.475; CI: 1.163, 5.266; p = 0.019 respectively).

**Table 7:** Final multivariate regression models of weight and length at different ages (Months) with Independent variables.

Independent Variables	Weight (kg) of study infants			Length (cm) of study infants		
	18 months	21 months	24 months	18 months	21 months	24 months
Maternal weight	0.031*** (0.015,0.047)	0.036*** (0.020,0.051)	0.028*** (0.014,0.043)	0.075** (0.023,0.126)	NS	NS
Maternal Height	NS	NS	NS	NS	0.114*** (0.047,0.182)	0.107*** (0.047,0.168)
Gestational Age	0.203** (0.023,0.383)	0.270** (0.094,0.445)	0.285*** (0.114,0.456)	NS	NS	NS
Father's education	NS	NS	NS	0.573** (0.166,0.979)	0.559* (0.127,0.990)	0.617** (0.206,1.027)
Zinc and placebo groups	NS	0.244* (468, 0.019)	NS	NS	NS	NS
Diarrheal duration	−0.027* (−0.046, −0.007)	NS	NS	−0.064* (−0.129, −0.001)	NS	NS
R^2^(%)	10.1	7.5	9.9	6.9	6.7	6.7

Values are partial regression coefficients.

Figures in parentheses indicate 95 % Confidence interval.

* p<0.05;**p<0.01;***p<0.001.

NS: not significant.

### Effect of zinc on diarrhea and respiratory infections

Median (range) number of episodes of diarrhea was 1.4 (0.0, 10.5) and 1.4 (CI 0.0, 5.6) per child per 100 days follow up in intervention and placebo groups respectively. Similarly, median (range) diarrheal duration was 6.1 (0.0, 98.6) and 7.0 (0.0, 32.9) days in zinc supplemented and control groups respectively per 100 days follow up (p = 0.10, Man–Whitney ‘U’ test). Median (range) number of episodes of respiratory infection was 0.7 (0, 4.4) and 0.9 (0.0, 4.9) respectively and median (range) duration of respiratory infection was 13.1 (0, 13.9) and 13.6 days (0, 46.0) respectively in zinc supplemented and placebo children for 100 days follow up. Both, episodes and duration of both infections were not significantly different in zinc or placebo groups.

Mean hemoglobin, serum zinc, copper and vitamin A levels were similar between groups; however, zinc deficiency (serum zinc <60 µg/dl) was prevalent in lesser proportion (26.5%) of zinc supplemented children compared to placebo group (44.1%) (Table 8).

**Table 8:** Biochemical parameters at 18 months of age.

	Zinc (n = 34)	Placebo (control) (n = 34)	P value
Hemoglobin (g/dl)	8.4±1.41	8.63±1.56	0.62
Zinc (µg/dl)	70.7±15.32	67.4±17.12	0.40
Vitamin A (µg/dl)	24.2±7.69	24.2±7.62	0.98
Copper (µg/dl)	151.4±25.38	158.6±28.60	0.28

All values are mean ± SD.

n =  Number of subjects.

## Discussion

Zinc supplementation for a mean period of 190 days (4 months upto 18 months of age), in full term normal infants, consuming an average of 590 Kcal/day, had significant effect on the skin fold thickness and wasting, but had no effect on their linear growth. Multiple linear regression models demonstrated maternal weight as a strong predictor of infant's weight and length at 18 months of age; and as expected, diarrhea duration had an adverse impact on infant's linear growth and weight gain.

Earlier, some studies have shown an increment in linear growth in stunted children or in children with low serum zinc at baseline, while some other studies on healthy term infants failed to show any beneficial effect of zinc on linear growth except for some effect on weight gain and cognitive function [10], [14], [15], [16]. In our study, blood collection was not done at the baseline and therefore interpretation of data with reference to baseline zinc status was not feasible. Our study population was very unique and that way may not be comparable to the above mentioned studies. For instance, all the children in our study were receiving cereal based complementary food that was inadequate in calories and micronutrients; and moreover, faltering in length appeared from 3 months of age onwards. Though most studies attributed the beneficial effect of zinc on linear growth to reduction in infections, improvement in appetite or replenishment of zinc status, information was lacking on whether the complementary foods given were adequate in zinc or whether they contained high phytate or not [10], [14], [15], [16]. It may be assumed that the infants in these studies received an adequate amount of complementary food with inadequate zinc and therefore supplementation of zinc must have contributed to full expression of growth potential [10], [14], [15], [16]. A possibility of bias also cannot be ruled out in one study, which registered high attrition in the zinc supplemented group [17]. In our study zinc supplementation increased fat fold (skin fold) thickness, though increment in linear growth was not observed. Dietary recall showed that the intakes were qualitatively and quantitatively insufficient in terms of energy and protein. Primarily, the diet was cereal based refined carbohydrates, which was low in protein as well as in micronutrients. Clearly, the huge calorie gap and low protein intake would have contributed to the lack of effect of zinc on linear growth of the children in our study.

Heinig et al could not demonstrate any effect of zinc on linear growth in infants receiving adequate energy with appropriate zinc, suggesting that additional zinc would not have effect on growth if adequate intake of balanced diet is ensured [18]. Lack of effect of zinc on linear growth in our study is in contrast with generally observed positive effect of zinc supplementation on breast fed infants in developing countries, suggesting that single nutrient supplementation, on a background of low calorie diet will not be adequate to meet the requirement for linear growth [18]. Furthermore, zinc is known to stimulate the synthesis of IGF-1 which may lead to pre-adipocyte differentiation [19]. In the present study, the significant increase in the TSF and SSF thickness from 18 to 24 months perhaps could be attributed to such an effect. Similar findings were observed in a study from Africa, which showed an increase in skin fold thickness after zinc supplementation in pre-adolescent children [20].

During 6 to 24 months of age, a child normally doubles in weight, and by 24 months almost attains half of that of adult height. But in contrast, the infants in the current study gained significantly lesser height and weight compared to the standard [21]. The onset of growth faltering was evident, from 3 months onwards, on par with the global data [22]. However, children in the current study continued to gain lesser weight for age and weight for length for a much longer period in contrast to the global data [22]. For instance, mean weight for age ‘z’ scores at 3 months was 0.95, which decreased progressively to –1.64 by 18 months and continued to decease to −1.69 by 24 months. ‘z’ scores for length (HAZ) over a period of 3 to 24 months also showed a similar trend. Moreover, unlike the global data and much like the data from other parts of India, the current study demonstrated faltering in length as well from 3 months of age [22], [3], [5], [23].

Growth faltering has multi-factorial etiology including poor nutrition and illness, in addition to other factors such as maternal weight and pregnancy weight gain. In the current study, multiple factors such as poor nutritional status of mothers, inadequate complementary food and diarrheal infections contributed significantly to poor growth in children. Maternal weight emerged as a predictor of gain in weight and length in infants at 18 months of age. At 21 and 24 months of age, while maternal weight predicted weight gain, maternal height predicted infant's length. Moreover, maternal weight and height had a protective effect on stunting at 21 months and 24 months of age respectively. Accordingly, it is credible to believe that early onset of stunting may have its antecedents in preconception and pregnancy [24]. Mother's weight at conception and subsequent gain during pregnancy are powerful determinants of an infant's size at birth and during the first six months of its life. The findings in the current study highlight the need for interventions in pre-conception, prenatal and early life to prevent growth failure in children in India.

While the RDA recommendation for 1–3 year old children is 1200 Kcal, the children in the current study were consuming only half that amount, similar to the National Nutrition Monitoring Bureau (NNMB) data for 1–3 year old children with rural background, indicating a huge gap in energy intake [25], [26]. Several studies have earlier recognized safe and adequate complementary feeding of breast fed infants as a critical factor in preventing growth failure [26], [27], [28]. An intervention study conducted by Bhandari et al that evaluated the effect of micronutrient fortified complementary feed and nutrition counseling on growth and morbidity failed to show any effect on linear growth except for a modest effect on weight gain [26]. The authors speculated lack of effect due to increased inter-current infections in the micronutrient fortified complementary feed group [26]. Similarly, in our study also diarrhea duration appeared to be playing a significant role in limiting the growth of the children, thus emphasizing the need to provide adequate complementary feeding under hygienic condition.

The current study was not powered to detect effect of zinc on diarrhea or respiratory morbidities and hence, we could not interpret on the effect of zinc on episodes of infections.

The compliance to supplementation in our study was similar to the Pemba study [29], where the duration of effective supplementation was 484.7 days (SD 306.6) out of the total supplementation duration of around 1000 days.

Optimal energy is essential for full growth potential. As stated earlier, the NNMB data for rural Indian children of 1–3 years of age shows 719 cal/day against the RDA of around 1200 calories for 1–3 year old children [25]. Inadequate calorie intake in the present study must have marred any primary growth promoting effect of zinc in the children. In addition, riboflavin, which was used as a placebo, is known to facilitate the absorption of minerals through cellular transport in gastrointestinal tract resulting in increased absorption of zinc and iron [30], [31]. Any enhanced fractional absorption of zinc in control children by riboflavin in the present study might have nullified the effect of zinc in the intervention group [30], [31], [32]. Yet another reason for lack of growth promoting effect of zinc could have been due to prompt treatment of infections. Children in the current study were regularly monitored and all the inter-current infections were promptly treated. As one of the effects of zinc on growth is through prevention/reduction of morbidity, prompt treatment of inter-current infections in both the groups might have masked any effect of zinc on growth [26]. Nevertheless, we believe that the strengths of this study lie in the setting and design. The study was a large double blind, population-based prospective study with an age and sex-matched control group. Moreover, the population studied is representative of majority of children in India, thus making the results generalizable.

In a nutshell, it can be safely concluded that most breast fed infants in relatively poor communities are inadequately fed and in such a scenario, a single micronutrient supplementation, such as zinc, without sufficient calories will not have any impact on the growth potential of the children. It is sad to note that a majority of urban dwelling children have poor growth spurt throughout the first 2 years of life. In such a situation, intervention through food supplementation is the need of the hour for women in the low socioeconomic urban community. This should begin from pre-conception and must be continued throughout pregnancy to postnatal periods.

## Supporting Information

Checklist S1 **CONSORT Checklist.**
(DOC)

Protocol S1 **Trial Protocol.**
(DOCX)
