# Supplementary material for: Effectiveness of Zinc Supplementation to Full Term Normal Infants: A Community Based Double Blind, Randomized, Controlled, Clinical Trial
Source: PLoS One. 2013 May 30;8(5):e61486. doi: 10.1371/journal.pone.0061486 (PMC3667840; doi:10.1371/journal.pone.0061486)
Supplement: Protocol S1 — Trial Protocol. (DOCX) [file pone.0061486.s002.docx]

**TRAIL PROTOCOL**

**Effectiveness of zinc supplementation to full term normal infants: A community based Double Blind, Randomized, controlled, clinical trial**

Protocol Number: NIN-ZINC-03 dt. March 31, 2003

Sponsor Nil

Principal Investigator Dr. K.V. Radhakrishna

Scientist “D”

National Institute of Nutrition

Indian Council of Medical Research

Jamai Osmania (PO), Hyderabad – 500007

Andhra Pradesh, India

Ph:040-27191254, Fax No:040-27019074

Email:  [vijrkk@yahoo.com](mailto:vijrkk@yahoo.com)

Study Manager Dr. R. Hemalatha

Scientist “E”

National Institute of Nutrition

Indian Council of Medical Research (ICMR)

Jamai Osmania (PO), Hyderabad – 500007

Andhra Pradesh, India

Ph:040-27191297, Fax No:040-27019074

# Email: [rhemalathanin@yahoo.com](mailto:rhemalathanin@yahoo.com)

Co-Investigators Dr. R. Hemalatha

Dr. J.J.Babu Geddam

Mr.P. Ajey Kumar

Dr. N. Balakrishna

Dr. Veena Shatrugna

**SIGNATURE**

Approved by

_______________________________ ______________

Dr. R. Hemalatha, MD Date

Scientist “E”

National Institute of Nutrition (ICMR)

Jamai Osmania (PO), Hyderabad – 500007

Andhra Pradesh, India

I have carefully read this protocol, and I agree to conduct the study according to protocol specifications and in compliance with the ICH GCP guidelines, the Declaratin of Helsinki and after having obtained the approval fdrom the Ethics Comitteee and the consent in writing from the patients

_______________________ ______________________________ _________

Co-Investigators’s Singature Name and Title Date

_______________________ ______________________________ _________

Co-Investigators’s Singature Name and Title Date

_______________________ ______________________________ _________

Co-Investigators’s Singature Name and Title Date

_______________________ ______________________________ _________

Co-Investigators’s Singature Name and Title Date

#### Form I, at the time of recruitment, ( At birth )

**(Double blind placebo controlled RCT of Zinc supplementation )**

1. **Date of Delivery**
2. **Date of Recruitment**
3. **Place of Delivery 1. Home, 2. Hospital, 3. Others**

**4. Id. No.**

**5. Sex 1. Male 2. Female**

1. **Mother’s Name………………………………………**
2. **Age**

**8. Mother’s Education 1. illit, .2. Primary, 3. Secondary, 4 degree**

**9. Occupation 1. UE, 2. DW, 3. Agri, 4 Se, 5. Sal**

**10. Obstetric history G……..P………A……..L……**

1. **Number of antenatal visits**
2. **Vitamin supple. Duration**
3. **Iron supple. Duration**

**14. Gestational age.**

**15. Delivery details 1. ND / 2. LSCS / 3. Forceps**

**16. Father’s name…………………………………………**

**17. Age**

**18. Fathers Education 1. illit, .2. Primary, 3. Secondary, 4 degree**

**19. Occupation 1. UE, 2. DW, 3. Agri, 4 Se, 5. Sal**

1. **No. of family members**

**21. Birth weight ( grams )**

**22. Maternal weight ( Kgs. )**

**23. Maternal height (Cms.)**

#### Zinc Study Form II Recall Morbidity data (15 day recall)

**Id. No……….**

| **Months**  **-------------parameter** | **1** | **2** | **3** | **4** | **5** | **6** | **7** | **8** | **9** | **10** | **11** | **12** |
| --- | --- | --- | --- | --- | --- | --- | --- | --- | --- | --- | --- | --- |
| **Present Illness** |  |  |  |  |  |  |  |  |  |  |  |  |
| **Diarrhea**  **(No. Episodes)**  **In last 15 days** |  |  |  |  |  |  |  |  |  |  |  |  |
| **Type of**  **Diarrhea** |  |  |  |  |  |  |  |  |  |  |  |  |
| Diarrhea**Frequency** |  |  |  |  |  |  |  |  |  |  |  |  |
| **Duration of Diarrhea** |  |  |  |  |  |  |  |  |  |  |  |  |
| **Admission**  **1. yes, 2. No** |  |  |  |  |  |  |  |  |  |  |  |  |
| **Treatment**  **Diarrhea** |  |  |  |  |  |  |  |  |  |  |  |  |
| **Cough + fever**  **No. of episodes**  **In last 15 days** |  |  |  |  |  |  |  |  |  |  |  |  |
| **Cough + Fever**  **Number days** |  |  |  |  |  |  |  |  |  |  |  |  |
| **Admission**  **1. yes, 2 No,** |  |  |  |  |  |  |  |  |  |  |  |  |
| **LRI**  **1.present**  **2. Absent** |  |  |  |  |  |  |  |  |  |  |  |  |
| **Investigations** |  |  |  |  |  |  |  |  |  |  |  |  |
| **Treatment Resp.** |  |  |  |  |  |  |  |  |  |  |  |  |

Present Illness 1. Diarrhea, 2. Cough, 3. Fever, 4. Cough + Fever

5. Dia + Fever 6. Cough + Fever + Dia 7. Nil, 9. NA

Diarrhea type 1. Watery 2. Mucus & Blood 3. Mixed type 4. Small frequent

Treatment diarrhes 1. ORS, 2. ORS + Antibiotic, 3. Antibiotics + I.V.fluids + im AB

4. sporolac 5. Nil

Invstigations done 1. Blood 2. CXR 3. Any other entered separately

4. Nil

Treatment Resp. Inf 1. Cough syrup / symptomatic, 2. Oral Antibiotic,

3. IM / I.V. antibiotic 4. Nil 5 .AB +Bronchodilators

#### Form II Morbidity data

(Double **blind placebo controlled RCT of Zinc supplementation)**

**Id. No……….**

| **Months**  **Parameter** | **1** | **2** | **3** | **4** | **5** | **6** | **7** | **8** | **9** | **10** | **11** | **12** |
| --- | --- | --- | --- | --- | --- | --- | --- | --- | --- | --- | --- | --- |
| **Deficiency**  **Signs** |  |  |  |  |  |  |  |  |  |  |  |  |
| **Feeding** |  |  |  |  |  |  |  |  |  |  |  |  |
| **Type of solid food** |  |  |  |  |  |  |  |  |  |  |  |  |
| **Routine**  **Supplementation** |  |  |  |  |  |  |  |  |  |  |  |  |
| **Compliance of**  **Supplementation**  **Days** |  |  |  |  |  |  |  |  |  |  |  |  |
| **Immunization** |  |  |  |  |  |  |  |  |  |  |  |  |
| **Others** |  |  |  |  |  |  |  |  |  |  |  |  |

Deficiency signs 1. B.Complex def., Stomatitis 2. VAD

Feeding details: 1. Exclusive Breast feeding, 2. BF plus water, 3. Mixed feeding

4. Top feeding 5. partially Weaned 6. Completely weaned

Solid food 1. Biscuits 2. Fruits, banana, 3. Rice etc. 4. Khichri

5. Eggs 6. Roti / Bread 7. Commercial foods

8. All Foods

# Routine 1. B complex & MVT 2 Zinc +BC 3. Iron

# 4. Vit A Supplementation 5. All + / Vit C 6. Calcium

# 7. All

Immunization: 1. OPV, 2. OPV, BCG 3. OPV, DPT , BCG

4. OPV, DPT, 5. OPV, DPT , Hep B, 6. Measles

7. MMR

## Others.: 1. Skin Infection, 2. CSOM, 3. Worm infestations / pica

4. Oral Thrush 5. Conjunctivitis

Form III Anthropometry data

(**Double blind placebo controlled RCT of Zinc supplementation)**

**Id. No _____**

| **Months**  **-------------parameter** | **0** | **3** | **6** | **9** | **12** |
| --- | --- | --- | --- | --- | --- |
| **Date of Visit** |  |  |  |  |  |
| **Weight**  **( Kgs)** |  |  |  |  |  |
| **Length**  **( CH )**  **(Cms)** |  |  |  |  |  |
| **H.C.**  **(Cms)** |  |  |  |  |  |
| **Crown rump length** |  |  |  |  |  |
| **Chest circum-**  **Ference** |  |  |  |  |  |
| **Sub scapular**  **skin fold** |  |  |  |  |  |
| **Upper arm**  **Length** |  |  |  |  |  |
| **Lower arm length** |  |  |  |  |  |
| **Mid arm circumference** |  |  |  |  |  |
| **Triceps skin fold** |  |  |  |  |  |
| **Mid thigh circumference** |  |  |  |  |  |
| **Calf circumference** |  |  |  |  |  |
| **Marternal Wt.** |  |  |  |  |  |

#### (Zinc study) Form II Recall Morbidity data(15 day recall)

#### Id. No……….

| **Months**  **-------------parameter** | **13** | **14** | **15** | **16** | **17** | **18** | **19** | **20** | **21** | **22** | **23** | **24** |
| --- | --- | --- | --- | --- | --- | --- | --- | --- | --- | --- | --- | --- |
| **Present Illness** |  |  |  |  |  |  |  |  |  |  |  |  |
| **Diarrhea**  **(No. Episodes)**  **In last 15 days** |  |  |  |  |  |  |  |  |  |  |  |  |
| **Type of**  **Diarrhea** |  |  |  |  |  |  |  |  |  |  |  |  |
| DiarrheaFrequency |  |  |  |  |  |  |  |  |  |  |  |  |
| Duration of Diarrhea |  |  |  |  |  |  |  |  |  |  |  |  |
| **Admission**  **1. yes, 2. No** |  |  |  |  |  |  |  |  |  |  |  |  |
| **Treatment**  **Diarrhea** |  |  |  |  |  |  |  |  |  |  |  |  |
| **Cough + fever**  **No of episodes**  **In last 15 days** |  |  |  |  |  |  |  |  |  |  |  |  |
| **Cough + Fever**  **Number days** |  |  |  |  |  |  |  |  |  |  |  |  |
| **Admission**  **1. yes, 2 No,** |  |  |  |  |  |  |  |  |  |  |  |  |
| **LRI**  **1. Present**  **2. Absent** |  |  |  |  |  |  |  |  |  |  |  |  |
| **Investigations** |  |  |  |  |  |  |  |  |  |  |  |  |
| **Treatment Resp.** |  |  |  |  |  |  |  |  |  |  |  |  |

Present Illness 1. Diarrhea, 2. Cough, 3. Fever, 4. Cough + Fever

5. Dia + Fever 6. Cough + Fever + Dia 7. Nil, 9. NA

Diarrhea type 1. Watery 2. Mucus & Blood 3. Mixed type 4. Small frequent

Treatment diarrhes 1. ORS, 2. ORS + Antibiotic, 3. Antibiotics + I.V.fluids + im AB 4. sporolac 5. Nil

Invstigations done 1. Blood 2. CXR 3. Any other entered separately 4. Nil

Treatment Resp. inf 1. Cough syrup / symptomatic, 2. Oral Antibiotic,

3. IM / I.V. antibiotic 4. Nil 5. AB + bronchodilators

#### Form II Morbidity data

(Double **blind placebo controlled RCT of Zinc supplementation )**

**Id. No……….**

| **Months**  **Parameter** | **13** | **14** | **15** | **16** | **17** | **18** | **19** | **20** | **21** | **22** | **23** | **24** |
| --- | --- | --- | --- | --- | --- | --- | --- | --- | --- | --- | --- | --- |
| **Deficiency**  **Signs** |  |  |  |  |  |  |  |  |  |  |  |  |
| **Feeding** |  |  |  |  |  |  |  |  |  |  |  |  |
| **Type of solid food** |  |  |  |  |  |  |  |  |  |  |  |  |
| **Routine**  **Supplementation** |  |  |  |  |  |  |  |  |  |  |  |  |
| **Compliance of**  **Supplementation**  **Days** |  |  |  |  |  |  |  |  |  |  |  |  |
| **Immunization** |  |  |  |  |  |  |  |  |  |  |  |  |
| **Others** |  |  |  |  |  |  |  |  |  |  |  |  |

Deficiency signs 1. B.Complex def., Stomatitis 2. VAD

Feeding details: 1. Exclusive Breast feeding, 2. BF plus water, 3. Mixed feeding

4. Top feeding 5. partially Weaned 6. Completely weaned

Solid food 1. Biscuits 2. Fruits, banana, 3. Rice etc. 4. Khichri

5. Eggs 6. Roti / Bread 7. Commercial foods

8. All Foods

# Routine 1. B complex & MVT 2 Zinc 3. Iron

# 4. Vit A Supplementation 5. All +/ Vit C 6. Calcium

# 7. All

Immunization: 1. OPV, 2. OPV, BCG 3. OPV, DPT , BCG

4. OPV, DPT, 5. OPV, DPT , Hep B, 6. Measles

7. MMR

## Others.: 1. Skin Infection, 2. CSOM, 3. Worm infestations / pica

4. Oral Thrush 5. Conjunctivitis

Form III Anthropometry data

(Double **blind placebo controlled RCT of Zinc supplementation)**

**Id. No……………….. Name……………………………….sex……B.Wt……**

| **Months**  **-------------parameter** | **15** | **18** | **21** | **24** |  |
| --- | --- | --- | --- | --- | --- |
| **Date of Visit** |  |  |  |  |  |
| **Weight**  **( Kgs)** |  |  |  |  |  |
| **Length**  **( CH )**  **(Cms)** |  |  |  |  |  |
| **H.C.**  **(Cms)** |  |  |  |  |  |
| **Crown rump length** |  |  |  |  |  |
| **Chest circum-**  **Ference** |  |  |  |  |  |
| **Sub scapular**  **skin fold** |  |  |  |  |  |
| **Upper arm**  **Length** |  |  |  |  |  |
| **Lower arm length** |  |  |  |  |  |
| **Mid arm circumference** |  |  |  |  |  |
| **Triceps skin fold** |  |  |  |  |  |
| **Mid thigh circumference** |  |  |  |  |  |
| **Calf circumference** |  |  |  |  |  |
| **Marternal Wt.** |  |  |  |  |  |

**Title of the Project :** Double Blind, Randomized, controlled, clinical trial of Zinc supplementation to full term infants.

**Investigators :** K. V. RadhaKrishna, R. Hemalatha, J.J. Babu,

N. Bala Krishna, P.Ajeya Kumar, Veena Shatrugna

**Duration :** 4 years

**Date of Initiation :** October 2003

**Budget :** 50,000=00 (2003)

**Brief outline**

Stunting is a major health problem in infancy and Preschool children in India, with mild to moderate stunting reaching almost 50% in some areas and severe stunting around 30%. Zinc deficiency is associated with primary growth failure and increased morbidity. Breast milk zinc, liver stores and plasma levels decrease by 4-6 months. However, 6-24 months of age is the critical period of growth and development in life and zinc requirements when expressed as per kg body weight are also high in preschoolers. Indian weaning and complimentary foods are not only low in zinc but also of low bioavailabilty, as the diet is cereal based with a high phytate content. In addition, infections specially diarrheal and respiratory are also high in these age groups. All these contribute to zinc deficiency state. Zinc is essential for lean body mass synthesis and zinc deficiency might lead to altered body composition.

Because of the homeostatic mechanism, plasma zinc levels are maintained but l

inear growth is affected even before reductions in serum zinc concentrations.

Acceleration of growth following zinc supplementation is considered as direct evidence of zinc deficiency.

Though severe zinc deficiency is not common in India, mild to moderate zinc deficiency could be highly prevalent due to low dietary intake and low bio availability of zinc because of high fiber and phytates in diet in predominanatly vegetarian diets. Zinc deficiency perhaps is reflected in the prevalence of low height for age (stunting) and low weight for age.

Zinc supplementation studies done in India and elsewhere have shown 25-30% reduction in diarrheal morbidity (episodes and duration) and 41% reduction in lower respiratory tract infections (Sazawal et al 1999, India). Since respiratory and diarrheal infections are one of the main contributors of infant mortality Zinc adequacy might help in reducing the IMR.

With this background it was hypothesized that even in full term infants, zinc supplementation initiated after the age of 4 months should reduce the morbidity, promote growth both ponderal and linear and prevent stunting and help in attaining optimum body composition.

It was decided to supplement elemental Zinc, through a double blind randomized controlled clinical trial, 5 mg/day, as zinc sulfate (10mg/ml), in syrupy base with Riboflavine 0.5 mg/day, and control children will receive only Riboflavine 0.5 mg / day, since Riboflavine is not known to have significant effects on growth or morbidity and also for ethical considerations.

Sample size of 268 (134 in zinc and 134 in control groups ) was calculated with the prevalence of severe stunting as 30% at 2 years based on national family health survey-2, and aiming at reduction of 50% in zinc supplemented group at 0.05 significance level, with a power of 80%, with a corrected chi-squared test. Assuming 20% dropouts to followup, the total sample required is 320. Assuming a 30% prevalence of 30% it is required to recruit around 420 full term infants to get a sample size of 320 FT normal infants.

**Aims & Objectives**

1. To Supplement Zinc ( as elemental zinc 5 mg / day ) plus Riboflavine 0.5 mg / day, to full term infants from 4 months to 18 months, and Riboflavine 0.5 mg / day, both in syrupy base to control group children.

2. To record Anthropometric data such as height, weight and skinfold thickness and other measures for body composition assessment at recruitment, and every 3 months till the end of the study.

3. To see whether the effects of supplementation persists even after the discontinuation of the supplementation for another 6 months i.e., till the age of 24 months..

4. Morbidity data will be collected by 15 day recall, and data regarding feeding practices and any other supplementation will be recorded for all the children.

**Study design**

Randomized, double blind, placebo controlled trial will be conducted in an urban slum (Addagutta). Inclusion criteria: term healthy infant without any obvious congenital anomalies and planning to stay in the study area. Preterm deliveries and infants with congenital abnormalities or birth trauma will be excluded from the study.

# Sample size

Taking the prevalence of severe stunting as 30% at 2 years as per national family health survey (NFHS-2) and aiming at reduction of stunting by 50% in supplemented group with 80% power at 0.05 significance level with a corrected chi-squared test and expecting dropouts at 20% the total sample size was arrived at 320 full time normal infants. Assuming 30% prevalence of low birth weight around in 20 full time infants need to be recruited.

# Data Collection

Field investigators will be trained to collect morbidity and anthropometric data of infants. Quality control testing for interrator reliability and reproducibility will be done every three months. The infants will be followed for atleast two years and data regarding growth, morbidity and feeding practices will be recorded. Morbidity data will be collected by 15 day recall method. Passing of more than 3 stools in 24 hours or stools with altered consistency, with or without mucus or blood or loose watery stools will be considered as diarrhea. Diarrheal episodes separated by 3 symptom free days will be considered as separate episodes. Respiratory tract infections will be defined as cough or cold with fever and recurrence after 3 symptom free days will be considered as a separate episode. After clinical examination lower respiratory tract infections will be confirmed by radiology.

Birth weight and maternal weight will be recorded. Anthropometric data like weight, length, head circumference, chest circumference, Head circumference and mid upper arm circumference and skin fold thicknesses at triceps, biceps, subscapular area will be collected every three months. Weights will be measured using a portable seca digital weighing machine with a sensitivity of 100 grams and length will be measured using infanto-meters to the nearest of 1mm., skin fold thickness at biceps, triceps and subscapular will be measured using harpenden calipers.

Maternal weight and height will be recorded within 1 month after delivery as most of the pregnancy weight gain is lost by then and every 3 months along with the children’s anthropometry data collection.

**Subject recruitment and Supplementation**

Full term pregnant women willing t**o** participate in the study will be enrolled from Addagutta slum. Neonates will be assessed for eligibility after delivery after taking a written consent. The slum has access to a government medical center as well as a bi-weekly clinic run by NIN, at the community center. Attempts will be made not to interfere with the routine medical care provided by the local health authorities and private medical practitioners. At 3 to 4 months age the infants will be randomly divided into two groups. The control groups of children will receive only riboflavin 0.5mg per day, whereas the intervention group will receive 5 mg of zinc plus riboflavin 0.5mg per day. Supplementation of zinc will be initiated between 3 to 4 months of age and will be continued for atleast 12 months. Biological Evans Limited has agreed to supply Riboflavin 1mg/ml with or without 10mg zinc per ml in a syrup base. The Director who is not connected with the study will code the placebo and zinc syrups bottles supplied by Biological Events. Empty bottles will be collected to ensure compliance.

Zinc excretion in Breast milk is <2mg/day by 2-3 months post partum. As per WHO proposed safe upper limit of Zinc intake per day at 0.5 to 1 year of age 13 mg/day and 1 to 6 years of age 23 mg/day. At supplementation levels of 5 milligrams per day no toxic effects are reported and it will not interfere with the absorption and metabolism of other micronutrients. As there is no routine supplementation program of Zinc, control children are at no extra disadvantage compared to normal population.

Blood samples (3 ml) will be collected at the end of the supplementation from a sub sample of 70 children after supplementation for estimation of Hemoglobin, Zinc, Copper and Vitamin A. Dietary intakes will be calculated by 24hours recall method in a sub sample of 70 children at the end of the supplementation.

It was suggested during the review meeting to supplement all full term children and record data but, morbidity data may be excluded from analysis and instead the study should concentrate only on growth parameters. To do serum zinc and copper levels in a subsample from the study children after supplementation is stopped i.e., at the end of 18 months.

**Expected outcome**

The prevalence of severe stunting should be less in zinc supplemented children compared to children supplemented with placebo.

# INFORMED CONSENT TO PARTICIPATE IN RESEARCH STUDY

NATIONAL INSTITUTE OF NUTRITION , HYDERABAD

(Double blind placebo controlled RCT of Zinc supplementation to FT infants)

I father / mother of baby named ________________________________________________

Aged _______ R/o __________________________________________________________ hereby give my consent to take part in the study titled ‘Zinc supplementation to full term infants to prevent growth retardation’.

I understand that the purpose of the study is to study the effect of Zinc supplementation on growth of infants from 3 months of age to 18 months.

I understand that the duration of the study is for 2 years, and blood samples ( 3 ml. each time ) will be collected from my child at 18 months .

I was not put under any pressure by anybody nor any monetary consideration was offered to me for participating in the study.

I understand that I can with draw from the study at any point without giving any reasons and my withdrawal from the study will not affect the routine care given to my child.

I understand that for all practical purposes I may not gain anything by participating in the study though in the long run it may be beneficial to my child.

I have been informed that no side effects have been reported with the above supplementation at the doses used in the study, and I am free to ask for any information regarding the study and will be answered by the concerned paediatrician / investigator.

I was explained the study protocol in my own language and give my consent after full understanding of the same.

I have been given a copy of this consent form

Witness:

1. Signature or thumb impression

Father / Mother

Place : _____________ Place : _____________

Date : ______________ Date : ______________
